# Impact on and use of health services by international migrants: questionnaire survey of inner city London A&E attenders

**DOI:** 10.1186/1472-6963-6-153

**Published:** 2006-11-29

**Authors:** Sally Hargreaves, Jon S Friedland, Philip Gothard, Sonia Saxena, Hugh Millington, Joseph Eliahoo, Peter Le Feuvre, Alison Holmes

**Affiliations:** 1 International Health Unit, Department of Infectious Diseases and Immunity, Faculty of Medicine, Imperial College, Hammersmith Hospital Campus, London, W12 ONN, UK; 2 Department of Primary Care and Social Medicine, Charing Cross Campus, Imperial College, W6 8RP, UK; 3 Charing Cross Hospital, A&E Department, London, W6 8RF, UK; 4 Statistics Advisory Service, Imperial College, London, SW7 2AZ, UK; 5 Dover Health Centre, Dover, Kent CT16 1RH, UK

## Abstract

**Background:**

Changing immigration trends pose new challenges for the UK's open access health service and there is considerable speculation that migrants from resource-poor countries place a disproportionate burden on services. Data are needed to inform provision of services to migrant groups and to ensure their access to appropriate health care. We compared sociodemographic characteristics and impact of migrant groups and UK-born patients presenting to a hospital A&E/Walk-In Centre and prior use of community-based General Practitioner (GP) services.

**Methods:**

We administered an anonymous questionnaire survey of all presenting patients at an A&E/Walk-In Centre at an inner-city London hospital during a 1 month period. Questions related to nationality, immigration status, time in the UK, registration and use of GP services. We compared differences between groups using two-way tables by Chi-Square and Fisher's exact test. We used logistic regression modelling to quantify associations of explanatory variables and outcomes.

**Results:**

1611 of 3262 patients completed the survey (response rate 49.4%). 720 (44.7%) were overseas born, representing 87 nationalities, of whom 532 (73.9%) were new migrants to the UK (≤10 years). Overseas born were over-represented in comparison to local estimates (44.7% *vs *33.6%; p < 0.001; proportional difference 0.111 [95% CI 0.087–0.136]). Dominant immigration status' were: work permit (24.4%), EU citizens (21.5%), with only 21 (1.3%) political asylum seekers/refugees. 178 (11%) reported nationalities from refugee-generating countries (RGCs), eg, Somalia, who were less likely to speak English. Compared with RGCs, and after adjusting for age and sex, the Australians, New Zealanders, and South Africans (ANS group; OR 0.28 [95% CI 0.11 to 0.71]; p = 0.008) and the Other Migrant (OM) group comprising mainly Europeans (0.13 [0.06 to 0.30]; p = 0.000) were less likely to have GP registration and to have made prior contact with GPs, yet this did not affect mode of access to hospital services across groups nor delay access to care.

**Conclusion:**

Recently arrived migrants are a diverse and substantial group, of whom migrants from refugee-generating countries and asylum seekers comprise only a minority group. Service reorganisation to ensure improved access to community-based GPs and delivery of more appropriate care may lessen their impact on acute services.

## Background

Changing UK immigration and asylum trends – in particular a greater net inflow of migrants from New Commonwealth and non-Commonwealth Africa, Asia, and Latin America [1] – are posing new challenges for service providers [2], especially in inner-city London to which migration is greatest [3]. There has been considerable speculation that migrants place a disproportionate burden on health services [4], particularly migrants from resource-poor countries such as asylum seekers, but data are limited.

Unlike other countries, which control the level of free or indeed any access to health services by migrants [5,6] the UK operates a relatively open system in practice. Migrants in the UK for a settled purpose (6 months or more) are entitled to register permanently with a community GP free of charge, with GPs acting as gatekeepers to other NHS services through a process of referral. However, it is a system that has recently being called into question, with proposals to exclude non-eligible migrants, including failed asylum seekers and irregular migrants, from access to free GP services [7], and recent changes to the enforcement and scope of hospital charging procedures [8]. These changes have been criticised because they fail to consider the potential impact on vulnerable migrants who may be unable to pay for health care, and implications for key services such as A&E Departments [9].

Several factors may affect access to GP services [10-15]. Migrants from refugee-generating countries that face war, upheaval, and/or economic decline, who include asylum seekers and refugees, migrant workers, and undocumented migrants, are known to be a particularly vulnerable inner city population who may face barriers to accessing appropriate primary care stemming from communication problems, social isolation and economic hardship [10,14,15]. Indeed, any new arrival could have difficulties understanding how to register with a GP, contacting GPs out of hours, and may use alternative pathways to seeking care [13-15]. Some GPs, perhaps concerned about practice targets for health improvement [16], may be reluctant to register certain groups of temporary migrants as permanent or temporary patients or to consider them as private patients [9,17]. Restricted access to primary care has been associated with increased workloads at A&E Departments [14,18,19].

The impact on and use of health services by migrants need to be further explored to inform appropriate service delivery to these groups. We compared the sociodemographic characteristics and impact of migrant groups and UK-born patients presenting to an acute hospital service and prior use of GP services.

## Methods

### Data collection

The survey took place at an A&E/Walk-In Centre at an inner London teaching hospital in an area with a substantial migrant population and established ethnic minority communities [3,20]. It was carried out over a 1 month period during the busiest working hours (8 am to 10 pm) Monday to Saturday. Reception staff gave questionnaires to all patients presenting during the survey period. Staff recorded patient's date of birth and sex before handing it to patients. Staff stated clearly to patients that the survey was both voluntary and confidential; a statement which was also written in bold type at the top of every questionnaire.

Patients brought to this hospital by ambulance are admitted via a dedicated entrance and are often too ill to complete a questionnaire, and were therefore excluded from the study. Parents were asked to fill out the questionnaire on behalf of children; however walk-in paediatric cases are rarely accepted at this service due to the presence of a specialist paediatric A&E Department nearby. Completed surveys were placed in a locked box.

### The questionnaire

The questionnaire included items on the patient's self-reported nationality and place of birth, time in the UK, employment status, language proficiency, immigration status, permanent GP registration, and whether they had consulted a GP regarding their current illness prior to presentation.

To facilitate access by diverse groups, the questionnaire was available in major local languages: Arabic, English, Farsi, Hindi, Polish, and Somali.

### Data definitions and analysis

We assigned respondents to one of 4 nationality analysis categories. Group one comprised patients self-reporting their nationality as from a refugee-generating country (the RGC group). This list was compiled from 10 years of routinely collected Home Office data [21] on individual claims for political asylum from specifically named countries (Table 1). The second group were patients from a country within the British Isles – The United Kingdom of Great Britain and Northern Ireland and the Republic of Ireland (the UK/Irish group). The third group comprised Australians, New Zealanders, and South Africans; these nationalities are defined by the Office of National Statistics as the major Old Commonwealth Countries represented in gross in-migration into the UK [22]; they also form significant communities in the local survey area [3] and have similar demographic profiles in terms of age, sex, time in the UK so it was appropriate to considered them as a single group. The fourth group comprised all other presenting migrants (OM group), most of whom were Europeans.

We compared differences between groups using two-way tables by Chi-Square and Fisher's exact test. We used logistic regression modelling to quantify associations of explanatory variables and outcomes. Statistical significance was taken as a cut off of p < 0.05 throughout. All data were analysed using STATA (Version 8.2, Stata Corporation). The study was approved by the Hammersmith Hospital Research Ethics Committee.

## Results

During the survey period, 4252 individuals presented to the A&E/Walk-In Centre, of which 990 presented outside of the survey hours. Of the 3262 who presented during the survey times, 1611 completed the form (response rate 49.4%).

### Characteristics of sample

Respondents reported 87 different nationalities (Table 2). 761 (47.2%) of 1611 respondents reported that they had been born in the UK or Republic of Ireland; 720 (44.7%) reported that they were born overseas, significantly higher than local Census data reporting a 33.6% overseas born population (p < 0.001; proportional difference 0.111 [95% CI 0.087–0.136]) [3]. 746 (98.0%) of UK/Irish born had acquired UK/Republic of Ireland citizenship. Overseas born reported a diverse range of citizen and immigration status' (Table 3). The majority of overseas born were new migrants to the UK (≤10 years; 532 [73.9%]) of whom 308 (40.5%) of 720 had been in the UK for ≤2 years, 153 (20.1%) for 3–5 years, and 71 (9.3%) for 6–10 years. 220 (30.6%) of 720 had been in the UK for less than 12 months. 21 (1.3%) of 1611 reported an immigration status of political asylum seeker or refugee (15 men, 5 women, 1 unknown; mean age 28.82 years [SD 13.02]; mean length of time in the UK 3.72 years [SD 2.27]). 728 (45.2%) of 1611 reported currently residing in the hospital catchment area; 84.8% within the boundaries of the strategic health authority (North West Thames).

178 (11.0%) of 1611 were in the RGC group, which included Polish and Somali from among the top 10 presenting nationalities (Table 4). 860 (53.4%) were in the UK/Irish group and 219 (13.6%) in the ANS group. 234 (14.5%) were in the OM group, of which the majority were Europeans (142 [60.7%] of 234). The dominant nationalities in the OM group were Italians, French, Spanish, American, and Portuguese. 120 (7.4%) of respondents did not get assigned to any group as they did not respond to the question on nationality.

### Characteristics of main nationality groups

The UK/Irish group (OR 1.51 [1.09–2.11]; p = 0.013), the OM group (2.00 [1.32–3.02]; p = 0.001), and the ANS group (7.76 [4.55–13.2]; p < 0.001) were more likely to have a paid job than the RGC group (88.6% ANS were in paid employment). The UK/Irish group (OR 21.66 [11.52–40.71]; p < 0.001) and the ANS group (26.18 [7.96–86.10]; p < 0.001) were significantly more likely to report that they spoke English at home than the RGC group. Respondents in the OM group were more likely to speak English than the RGC group, but this difference was not statistically significance (1.51 [0.94–2.42]; p = 0.08).

#### (i) Registration with a permanent GP

Overall, 1108 (68.8%) of 1611 respondents had a permanent GP in the community, consistent with Trust data, but this differed significantly between groups ranging from 32.0% (ANS) to 87.1% (UK/Irish; Table 4). After adjusting for age and sex, there was no difference in GP registration between RGC group and UK/Irish (Table 5). However, the ANS and OM group were significantly less likely to have a permanent GP than the RGC group (ANS group: 0.26 [0.16–0.40], p < 0.001; OM group: 0.55 [0.36–0.84] p = 0.006). There was a high proportion of GP registration among the 21 respondents with political refugee or asylum seeker status (81.0%).

Independent factors associated with not having a permanent GP were: being under 35 years of age, being male, being from the ANS and OM group, and being a migrant in the UK for less than 5 years; table 5. After adjusting for age and sex, therefore, factors such as English language proficiency were not associated with barriers to GP registration. Lack of a GP did not impact on time to presentation to acute services among nationality groups. 1222 (83.3%) of 1467 respondents reported that they had been ill for ≤1 month before presentation and 242 (16.5%) for >1 month, with no significant differences noted across analysis groups.

#### (ii) Mode of access and prior GP consultation

The majority of respondents reported that they had self-referred to this service, with no differences noted across nationality groups (UK/Irish 74.7%; RGC 76.3%; OM 78.7%; ANS 78.4%). 223 (13.8%) of 1611 respondents reported being directly referred to the A&E/Walk-In Centre by the GP; 129 (8.0%) were referred by the nurse-led telephone advice service NHS Direct.

406 (25.2%) respondents reported that prior to attending this A&E/Walk-In Centre, they had prior consultation with a GP regarding the presenting illness. There was no evidence of a difference between the RGC and the UK/Irish group in terms of prior consultation with a GP (OR 0.82 [95% CI 0.58–1.16]; p = 0.263). However, the RGC group was significantly more likely to have had prior consultation with a GP about the current illness than the ANS group (OR 0.46 [0.29–0.74]; p = 0.001) or OM group (OR 0.42 [0.26–0.67]; p < 0.001).

## Discussion and conclusions

We found that international migrants, most of whom were recent arrivals to the UK, posed a significant workload to this service and were a diverse group. There were two distinct groups of migrants at this service. First, the dominant presence in the cohort of Australians, New Zealanders, and EU citizens, coming in on work and study visas, which reflects current national gross in-migration data [22]. These individuals were significantly less likely to be registered with GPs (ANS: 32% registered). Second, the smaller RGC group, who despite obvious barriers to care such as language, were significantly more likely to have GP registration (58%) and to have made prior contact with a GP about the current illness before attending the A&E/Walk-In Centre. Registration and/or contact with GP services did not influence mode of access to hospital services, with equal rates of self-referral to this A&E/Walk-In Centre across nationality groups, suggesting that there are other factors involved in use of health services that may differ between migrant groups.

Few studies have sought to gain numerical data on use of GP and acute services by recently arrived migrant groups, despite the need for evidence to inform service delivery at this time [7,8]. Our study is of interest to service providers as it considers the diversity of international migrants using this type of service. Additional workload in specifically London A&E Departments from temporary visitors such as tourists and commuters who present without GP registration has previously been described [23]; our survey has enabled us describe the impact on such services of a more diverse migrant population. Although these snap-shot surveys have limitations, our response rate was considered high in what is a difficult context to collect data and we are confident that these findings reflect the day-to-day experiences of this service and are likely to be generalisable to other inner-city London units. There are few studies in this area adopting a self-completion questionnaire approach that are useful for comparison. Not collecting data on migrants presenting via ambulance could have biased the results in favour of higher presentation rates of more affluent migrant groups (ANS or OM groups), though this is unlikely to have had a significant impact on key findings. The absence of interpreters in the department to administer questionnaires meant that some patients for whom a translated questionnaire was not available may have been excluded from the study. However, patients presenting to this service who did not speak English largely arrive with a friend or family member to interpret for them, and who we are aware supported patients in the completion of survey forms. In addition, we translated the questionnaires into the dominant languages of presenting patients to this hospital, so feel confident that we will have captured the majority of presenting migrants.

We and others report that only around 50% of the cohort resided within the hospital catchment area [24]; additionally, we found that overseas-born patients as a group are over-represented at the service in comparison to local data [3]. This over-representation likely reflects the large number of recent arrivals and temporary migrants using this service, who will not have been accounted for in local estimates. A&E Departments may be an additional attraction for migrants who may not be entitled to register with a GP free of charge, or may perceive that they are not entitled, because they are freely accessible to all and health staff will address both emergency and non-emergency health needs. One other national UK study at open access Genitourinary Medicine clinics reported an increase in workload from migrant populations comprising asylum seekers and irregular migrants [25], but did not consider other categories of migrants.

Patients from the ANS and OM group were younger, had higher socioeconomic status (paid jobs), and likely a better baseline health status – all of which are factors known to impact on mode of access to primary and secondary care [10-12]. Some may not have been entitled to register permanently with a GP because of their recent arrival or temporary status. This may be a specific issue for GPs in high migrant areas who are concerned about the impact of temporary migrants on practice targets; the new GP contract has largely removed financial incentives for GPs to register temporary patients [26]. We hypothesise that some patients from these groups could feasibly have been using this service as their source of primary care in the absence of GP registration, and that this could explain their high attendance. Previous studies have shown a range of rates of non-emergency attenders at UK A&E Departments (6%–80%), which is deemed detrimental as it adds to waiting times, degrades the quality of care such services can deliver, and increases costs [27]; however, none have addressed migration status. Although not advertised, the presence of GPs at the attached the Walk-In Centre at this survey site (patients are triaged to the Walk-In according to needs after initial registration at the A&E Department) could have attracted an additional number of migrants aware of this option. Walk-In Centres, designed to address issues of access and unmet need in primary care, have been shown previously to benefit mainly white, middle class patients in terms of improved access [28]; our data perhaps suggest that such GP-led services could disproportionately attract certain migrant groups with low GP registration rates.

GP registration rates had little bearing on subsequent mode of access, with equal rates of self-referral to this service across all groups, which suggests that accessing appropriate primary health care is more complex than finding and registering with a GP. Greater GP use among the RGC group, despite obvious barriers such as language, mirror recent studies investigating GP registration among refugees and other RGC migrants [14,29] and may reflect local initiatives to facilitate registration amongst minority groups. However studies show that additional barriers, associated with ethnicity and communication problems, may operate at the service provider level in terms of access to appropriate quality care to meet needs once inside the system [10,12]. Although it was outside of the scope of our survey to assess reason for attendance, these factors have previously been associated with increased use of acute services by more vulnerable migrant groups [14,18,19].

Our findings have implications for resource allocation in terms of enabling inner-city London services to cope with increasing rates of in-migration to the UK. Initiatives that encourage migrants with low registration rates to register with and use GPs could alleviate pressures on acute services. We need to assess how best to deliver appropriate quality care to more vulnerable groups beyond merely facilitating GP registration. Models of best practice, considering the approaches of other European countries [5], need to be explored to elucidate how best to deliver primary care to migrant groups, whilst consideration must be given to the impact of temporary migrants on GP practice targets and resources.

Recent attention on the impact of 'foreign born' patients on the NHS, and the tightening up of charging systems for non-eligible migrants [7,8], has not considered the diversity of this group. Focus on the burden of asylum seekers, particularly from resource-poor African countries, misses the significant number of migrants from wealthier countries (eg, USA, Europe) who may pose similar organisational and charging dilemmas. Our data suggest that recent proposals to exclude or charge non-eligible migrants at GP services [7,9] could have a detrimental impact on free A&E Departments in terms of an increase in presenting migrants.

## Competing interests

The author(s) declare that they have no competing interest.

## Authors' contributions

All authors were involved in the study design, and in the acquisition and interpretation of data. JE and SH performed the statistical analysis. SH, JSF, and AH drafted the final article and all authors were involved in revising it critically for important intellectual content. All authors read and approved the final manuscript.

## Pre-publication history

The pre-publication history for this paper can be accessed here:



## Figures and Tables

**Table 1:** Countries generating political asylum seekers and refugees to the UK in the past 10 years

Named countries by region*
**Europe** Albania, Czech Republic, Macedonia, Moldova, Poland, Romania, Russia, SAM+, Turkey, Ukraine
**Americas** Colombia, Ecuador, Jamaica
**Africa** Algeria, Angola, Burundi, Cameroon, Congo, Democratic Republic of Congo, Eritrea, Ethiopia, Gambia, Ghana, Ivory Coast, Kenya, Liberia, Nigeria, Rwanda, Sierra Leone, Somalia, Sudan, Tanzania, Uganda, Zimbabwe
**Middle East** Iran, Iraq
**Rest of Asia** Afghanistan, Bangladesh, China, India, Pakistan, Sri Lanka, Vietnam

*Source: Home Office data on individual claims for political asylum in the UK from specifically named countries only [21]. For the past 10 years the named countries generating political asylum seekers to the UK has remained unchanged, though the numbers arriving from each country have fluctuated.

+Serbia and Montenegro replaced the Federal Republic of Yugoslavia/FRY from Feb 5, 2003; SAM includes the Province of Kosovo.

**Table 2:** Self-reported nationality of presenting patients

**Self-reported nationality**	**Number of patients n (%)**
British/English/Irish/Northern Irish	860 (57.7)
Australian	126 (8.5)
Polish	59 (4.0)
New Zealander	49 (3.3)
South African	44 (3.0)
Italian	31 (2.1)
French	25 (1.7)
Spanish, American	21 (1.4)*
Somali	13 (0.9)
Portuguese, Japanese	12 (0.8)*
Brazilian, Iranian	10 (0.7)*
Canadian, Indian	9 (0.6)*
German	8 (0.5)
Czechoslovakian, Chinese, Dutch, Hungarian, Afghan	7 (0.5)*
Jamaican, Nigerian, Greek, Iraqi, "European"	6 (0.4)*
Filipino, Turkish	5 (0.3)*
Yugoslavian, Algerian, Mauritanian, Swedish, Moroccan	4 (0.3)*
Belgian, Colombian, Sri Lankan, Lithuanian, Ethiopian,	3 (0.2)*
Albanian, "African", Serbian, Swiss, Venezuelan, Gambian, Pakistani, Malaysian, Zimbabwean, Palestinian, Kosovan, Finnish, Korean, Bulgarian	2 (0.1)*
Cypriot, Ghanaian, "Asian", Bahamian, Kazak, Saudi Arabian, Sudanese, Indonesian, Syrian, Macedonian, Romanian, Jordanian, Nepalese, Grenadian, Tongan, Taiwanese, Austrian, Mongolian, Croatian, Egyptian, Ivorian, Barbadian, Maltese, Ecuadorian, Bangladeshi, Ukrainian, Thai, Angolan, Guyanese, Mexican, Zambian, Norwegian, Danish, Kurdish	1 (0.1)*
**Total named nationalities: 87**	**1491 (100%)$**

*Data are n (%), which equates to the number of patients from each named country in the first column. $ 120 of 1611 respondents did not answer this specific question.

Patients who reported their nationality non-specifically as "Asian" (n = 1), "African"(n = 2) or "European" (n = 6) were analysed in the OM group.

**Table 3:** Self-reported citizenship/immigration status

**Citizenship/immigration status**	**Total sample n (%)**	**Overseas born n (%)**
**Overall**	**1611 (100%)**	**720 (100%)**
UK/Republic of Ireland citizen	967 (59.8)	209 (29.0)
Work permit	176 (10.9)	176 (24.4)
EU citizen	161 (10.0)	155 (21.5)
Student visa	58 (3.6)	56 (7.8)
Tourist	35 (2.2)	34 (4.7)
Other*	34 (2.1)	33 (4.6)
Refugee and/or asylum seeker	21 (1.3)	21 (2.9)
Claiming citizenship	20 (1.2)	19 (2.6)
Business trip	8 (0.5)	7 (1.0)
No response	121 (7.5)	10 (1.4)

*6 of 34 patients who circled this category noted on the survey sheets that they were in the UK on ancestral visas or a permanent resident.

**Table 4:** Basic characteristics by nationality group

**Analysis group**	**N**	**Mean (years) age (SD)**	**Time (years) in UK (SD)**	**Male sex N (%)**	**N (%) speaking English at home**	**N (%) with a paid job**	**N (%) with a permanent GP**
Refugee-Generating Country (RGC) group+	178	32.19 (14.50)	6.21 (9.93)	94 (53.7%)	121 (73.33%)	93 (53.0%)	103 (57.9%)
UK/Irish group	860	37.66 (17.82)	26.23 (14.16)	387 (47.7%)	834 (98.34%)	528 (63.3%)	749 (87.1%)
Australia/New Zealand/South African (ANS) group	219	27.84 (10.21)	1.83 (5.11)	94 (42.9%)	216 (98.6%)	194 (88.6%)	70 (32.0%)
Other Migrant (OM) group	234	30.07 (10.79)	4.78 (8.33)	85 (38.6%)	186 (80.51%)	157 (69.5%)	110 (47.0%)

+ 175 (98.3%) of 178 reported that they were not born in the UK.

**Table 5:** Multivariate analysis: significant factors associated with having a permanent GP

**Characteristic**	**Adjusted odds Ratio (95% CI)**	**P value**
**Age**		
0–34 years*	1.00	
35+ years	3.11 (1.92–5.03)	<0.0001

**Sex**		
Male*	1.00	
Female	1.47 (1.00–2.27)	0.045

**Prior contact with a GP**		
No*	1.00	
Yes	3.24 (1.97–5.34)	<0.0001

**Nationality**		
RGC group*	1.00	
UK/Irish group	0.49 (0.19–1.3)	0.157
OM group	0.28 (0.11–0.71)	0.008
ANS group	0.13 (0.06–0.30)	<0.0001

**Time in the UK**		
0–5 years*	1.00	
6–10 years	2.59 (1.28–5.22)	0.008

*Baseline group.
